# Effects of Cervical Spine Mobilization on Respiratory Function and Cervical Angles of Stroke Patients: A Pilot Study

**DOI:** 10.3390/healthcare9040377

**Published:** 2021-03-29

**Authors:** Ho Jung An, Shin Jun Park

**Affiliations:** 1 Department of Physical Therapy, Dongnam Health University, 50, Cheoncheon-ro 74beon-gil, Jangan-gu, Suwon-si, Gyeonggi-do 16328, Korea; 0628jjang@hanmail.net; 2 Department of Physical Therapy, Suwon Women’s University, 1098, Juseok-ro, Bongdam-eup, Hwaseong-si, Gyeonggi-do 18333, Korea

**Keywords:** joint mobilization, FHP, cervical spine, stroke, MIP, CVA

## Abstract

The forward head posture (FHP) of stroke patients has a negative impact on respiratory function. Cervical spine mobilization is a manual therapy technique that used to prevent and treat FHP and respiratory function. This pilot study investigated whether cervical spine mobilization can effectively improve outcomes following FHP and respiratory function of stroke patients. Twenty-four patients participated in our assessor-blinded randomized controlled trial. All the participants received neurodevelopmental treatments (gait training and trunk rehabilitation). The experimental group additionally received 15-min sessions of cervical spine mobilization three times per week for 4 weeks. The control group received cervical spine sham mobilization during the same period. For the cervical angles, the cranial vertebral angle (CVA) and cranial rotation angle (CRA) were measured. A respiratory function test was performed to measure the forced vital capacity (FVC), forced expiratory volume in 1 s (FEV1), peak expiratory flow (PEF), maximal inspiratory pressure (MIP), maximal expiratory pressure (MEP), and chest circumferences (upper and lower chest sizes). Except for MIP, there was no significant difference between the experimental group and the control group. The CVA and CRA were significantly increased in the experimental group only. Cervical spine mobilization improved cervical angles and inspiratory function of the stroke patients in this study. However, a comparative study with a larger number of patients is needed to confirm this finding from our pilot study, which had a small sample size.

## 1. Introduction

Common stroke has a negative impact on patients’ voluntary respiration [1]. Muscle weakness is the most evident respiratory problem in stroke patients [2] and is associated with a reduction in pulmonary function, functional outcome, and postural control [3].

Altered postural control after a stroke reduces the patient’s ability to maintain postural alignment and results in a forward head posture (FHP). In general, an FHP causes respiratory muscle weakness [4] and reduces pulmonary function [5]. Improving the FHP of stroke patients can improve their respiratory functions [6]. For this reason, the FHP following a stroke must be corrected to improve the respiratory function [6]. Voluntary respiration refers to the process of forced inhalation and exhalation, and the smooth movement of the thoracic cage is necessary for deep breathing [7]. The FHP shortens the abdominal muscles, thereby reducing chest motion during breathing [8] and, consequently, pulmonary function [9].

Autonomic nervous system dysfunction can also reduce the respiratory function [10]. The FHP can affect the cervical region sensory motor control and autonomic nervous system [11]. Autonomic nervous system dysfunction has been observed in stroke patients [12].

In a study on cervical realignments, chin-tuck exercise increased the cervical flexor and extensor muscle strengths, positively affecting the pulmonary functions of stroke patients [6]. However, mobilization exercises are more effective than stabilization exercises such as the chin-tuck exercise for improving the FHP. Therefore, a mobilization-based intervention is needed to improve the FHP [13]. Spine mobilization is a manual technique that can reduce pain or increase the limited segmental motion [14]. Cervical spine mobilization has been shown to improve cervical alignments in stroke patients with FHP [15]. Stroke patients with swallowing dysfunction have reduced diaphragm excursion [16]. Cervical spine mobilization improved the cranial vertebral angle (CVA), craniocervical flexion, and swallowing function of stroke patients [17]. Furthermore, cervical and thoracic mobilizations for improving the FHP and thoracic kyphosis increased the forced vital capacity (FVC), forced expiratory volume in 1 s (FEV1), and peak expiratory flow (PEF) of stroke patients [18].

Cervical spine mobilization activates the autonomic nervous system, thereby increasing the respiratory function [19,20,21]. Despite all the research, no study has examined the relationship between the FHP and respiratory function of stroke patients after cervical spine mobilization. Thus, the present study examined the changes in respiratory function and cervical alignment after cervical mobilization in stroke patients.

## 2. Methods

### 2.1. Study Design and Process

This pilot study was conducted over 4 weeks. It is an accessor-blinded randomized controlled trial involving 24 stroke patients. A baseline test before the intervention and a post-intervention test 4 weeks after the baseline test (after 12 sessions) were conducted. Written consent was obtained from all the participants before the baseline test. The participants were randomly assigned to an experimental group (cervical spine mobilization) or a control group (sham spine mobilization) after completing the baseline test. Computer-generated block randomization with a 1:1 ratio between the groups was used. All the evaluators and participants were blinded to the group assignment. Only one physiotherapist who performed the spine mobilization was aware of the group assignment.

Twelve patients were assigned to each group, which is an appropriate sample size for a pilot study [22]. The experimental group (*n* = 12) received 20 min of neurodevelopmental treatment (NDT) and 10 min of cervical spine mobilization. The control group (*n* = 12) received 20 min of NDT and 10 min of sham spine mobilization. The 20 min of NDT program consisted of trunk rehabilitation. Trunk rehabilitation is a trunk control exercise for stability in three different planes (sagittal, frontal, and transverse). Each treatment session, thus, lasted 30 min and was held three times per week for 4 weeks for a total of 12 sessions. None of the participants dropped out of the study before the end of the 4-week intervention. This study was conducted in accordance with the Declaration of Helsinki and approved by the institutional review board of Yong-in University (approval No. 2009-HSR-198-2).

### 2.2. Participants

The inclusion criteria for the study participants were as follows: stroke disease duration of ≥6 months, voluntary participation, a Korean-Mini Mental State Examination score of ≥24 points, cervical anteroposition (CV angle < 49°) [23,24], no limited lip movement, hypomobility of the cervical spine during manual evaluation [14], no orthopedic surgery of the cervical spine, and no neurological diseases except stroke. Patients were excluded if they had a cardiac pulmonary or peripheral vascular disease, severe dizziness during pulmonary function tests, chest pain during chest circumference measurement, poor sitting balance, hemineglect or apraxia, or depressive disorders.

### 2.3. Intervention Methods

All the participants received five sessions of neurodevelopmental treatment (NDT) over 4 weeks, including gait training and trunk rehabilitation. The experimental and control groups additionally received cervical and sham spine mobilization therapies for 10 min each.

#### Cervical Spine Mobilization

One of the following joint mobilization techniques was applied on the cervical spine: lateral movements, posteroanterior central vertebral pressure, posteroanterior unilateral vertebral pressure, anteroposterior unilateral vertebral pressure, transverse vertebral pressure, and flexion. An active movement test was performed to examine the quality and amount of neck motion before choosing the mobilization technique. After the active movement test, passive physiological intervertebral movements (PPIVMs) and passive accessory intervertebral movements (PAIVMs) were performed to identify the sites of hypomobility where spine mobilization would be applied [25]. Studies have reported lower cervical flexion to be a more serious problem than the upper cervical extension in the FHP [26,27]. Therefore, posteroanterior central vertebral pressure was applied on the lower cervical spine (C7/T1 segment) if no hypomobility was detected [27]. In this study, four sets of Maitland joint mobilization grade III were performed, each lasting 1 min, with a 1-min break between the sets. Sham mobilization was performed at a random location in the cervical spine. For sham mobilization, only manual contact was made without any movement.

### 2.4. Assessments

A single physiotherapist with no involvement in this study performed all the assessments. The assessor was not informed about the hypothesis being tested in this study.

#### 2.4.1. Cervical Angles

The Windows version of ImageJ (National Institute of Health, USA) was used to measure the cervical angles [28]. The participants were seated in a comfortable and natural posture on a chair before the measurement and directed to stare at a mark in front of the chair. A measurement camera was placed 80 cm from the chair, with its lens at the level of the participants’ shoulders. The cranial rotation angle (CRA) and CVA were measured [29,30]. The CRA was measured at the intersection between the line connecting the tragus of the ear to the C7 spinous processes and the line connecting the tragus to the canthus of the eye. The CVA was measured at the intersection between the line joining the tragus and C7 spinous processes and the horizontal line passing through the C7 spinous processes.

#### 2.4.2. Pulmonary Function Test

A spirometer (Pony FX, COSMED Inc., Rome, Italy) was used to measure pulmonary function. The FVC, FEV1, and PEF were measured to assess the pulmonary function. The participants were seated on a chair during the measurement. A nose clip and mouthpiece were used to prevent air leakage. The mouthpiece was placed 2 cm into the mouth during the pulmonary function test. The participants performed respiration three times in a stable condition, followed by maximum possible inspiration. They then performed strong and quick expirations according to the verbal cues from the assessor. In the expiration phase, the participants were instructed to exhale for as long as possible (6 s). The pulmonary function test was performed three times, and the maximum measurement from the three tests was recorded. For the maximal inspiratory pressure (MIP) and maximal expiratory pressure (MEP) measurements, the participants repeated maximal inspiration and expiration until a cue, with a mouthpiece in their mouths and in the same posture as in the pulmonary function test. The maximum measurements from the three repeated tests were used.

#### 2.4.3. Chest Circumference

A measurement tape (Baseline 12-1201 Gulick, USA) was used to measure the chest circumferences around the upper and lower rib cages. The horizontal upper chest circumference was measured at the chest levels of the third intercostal space (ventral) and fifth thoracic spinous process (dorsal). The horizontal lower chest circumference was measured at the chest levels of the tip of the xiphoid process (ventral) and 10th thoracic spinous process (dorsal). The chest circumferences were measured after maximum inspiration and expiration. The difference between the two chest circumferences was used in the analysis [31].

### 2.5. Statistical Analyses

All statistical analyses were performed using the SPSS Ver. 21.0 software (SPSS Inc., Chicago, IL, USA). The chi-square distribution and Mann–Whitney *U* test were used to test for homogeneity. The Wilcoxon signed-ranked test was used to assess the changes from the baseline test and the post-intervention test performed 4 weeks later. The Mann–Whitney *U* test was used to compare the results between the experimental and control groups. The results of the baseline and post-intervention tests are presented as mean and standard deviation. The significance level alpha was set to 0.05 in this study.

## 3. Results

### 3.1. General Characteristics

Table 1 shows the general characteristics of the study participants. We found no significant differences in the general characteristics of the participants in the experimental and control groups.

### 3.2. Changes in the Cervical Angles

Only the experimental group showed a significant increase in the CVA and CRA. However, no significant differences were found between the experimental and control groups (Table 2).

### 3.3. Changes in Respiratory Function

Both the experimental and control groups showed significant increases in the FVC, FEV1, PEF, MIP, MEP, and upper and lower chest circumferences. Both groups showed no significant differences in the FVC, FEV1, PEF, MEP, and upper and lower chest circumferences. In addition, the experimental group displayed a significant increase in the MIP as compared with the control group (Table 3).

## 4. Discussion

The results of this study are consistent with those of a previous study in which cervical spine mobilization was applied in stroke patients [15]. The strength of this study is that it is the first to discover that cervical spine mobilization can effectively improve cervical alignment and inspiratory function of stroke patients with an FHP.

Secondary non-neural factors that arise as stroke patients entering the chronic phase after a stroke are a possible cause of the FHP of stroke patients [32,33,34,35]. In this study, the experimental group showed improvements in the CVA and CRA after spine mobilization. The spine mobilization technique used in this study was applied to areas of hypomobility. In other words, the mobilization technique was not applied to the same area for all the patients. A physiotherapist identified the limited segmental joint level of the cervical spine through a manual evaluation (segmental palpation) to determine the specific directions of the bony level for the mobilization technique. In this study, a single physiotherapist performed cervical spine mobilization to ensure the high reliability of the results. Joint mobilization has inter-treatment differences in the amount of force applied but has high intra-therapist repeatability [36,37]. In a previous study, the cervical spine mobilization technique used in our study increased the active cervical ranges of motion (flexion, extension, side flexion, and rotation) [25]. The increased cervical ranges of motion (flexion and rotation) were associated with a greater increase in the CVA [26]. In addition, posteroanterior cervical spine mobilization reduces the cervical stiffness to increase the active cervical range of motion [38]. The cervical spine range of motion is increased by arthrokinematic cervical spine motion. Spine mobilization increases the mobility of the cervical facet joint and can affect the clinical symptoms of pain [39]. Thus, the increase in the movement of the joint surfaces in the cervical spine appears to affect the motion of the arthrokinematics cervical spine and consequently improves the CVA and CRA.

Cervical spine mobilization exerts neurophysiological effects on the cervical muscle strength by activating the descending pathways from the periaqueductal gray area (PAG) of the midbrain, reducing superficial neck flexor muscle activity and increasing deck flexor muscle activity [40]. Cervical spine mobilization was found to immediately increase the muscle strength [41,42,43]. C5–C6 mobilization immediately increased the shoulder external rotator muscle strength [41], and C5–T1 mobilization increased the range of motion of elbow extension [42]. As the afferent sensory input improved by joint mobilization affects the efferent nerve activity at the cervical spine segmental level, joint mobilization can improve the muscle strength. The diaphragm, which is essential for voluntary respiration, is controlled by the phrenic nerve originating from the cervical roots, C3, C4, and C5 [44,45]. The FHP can reduce respiratory function by the entrapment of the phrenic nerve [46], and the third cervical spine mobilization immediately increases the MIP [43]. As cervical spine mobilization affects the afferent phrenic nerve activity, cervical spine mobilization appears to improve the MIP more significantly in the experimental group than in the control group in our study.

FHP in stroke patients is closely associated with thoracic kyphosis [32], and trunk rehabilitation may be used to improve thoracic kyphosis in stroke patients [47]. In this study, a trunk rehabilitation-based NDT program was applied to all subjects for postural control of stroke patients. Therefore, it is believed that there was no difference in cervical angle between the two groups because postural control due to trunk rehabilitation can improve body alignment.

Cervical and thoracic mobilizations improve the FVC, FEV1, and PEF of stroke patients by promoting thoracic movements [18]. In this study, joint mobilization was applied to the cervical spine only to examine the effect of cervical spine mobilization. The effect of joint mobilization on the chest circumference was not examined. Patients with FHP exhibited a reduction in the lower chest circumference but not in the upper chest circumference during respiration, possibly because of abdominal muscle shortening [8]. No significant differences in chest circumference were observed because cervical joint mobilization does not affect abdominal muscle flexibility. In our study, cervical spine mobilization did not improve pulmonary function possibly because the pulmonary function test used in this study assessed the maximum voluntary expiration. The maximum voluntary expiration entails the shortening of the abdominal muscles, and the FVC, PEF, and FEV1 are dependent on it. In a previous study, spine mobilization applied on the rib cage to increase the inspiratory capacity of stroke patients increased the inspiratory muscle activity but had no effect on pulmonary function [48]. This result supports the results of our study.

In conclusion, a trunk rehabilitation-based NDT program is important for cervical alignment and pulmonary function in stroke patients, and cervical mobilization can reinforce that effect. This study has several limitations. A major limitation is the small sample size. As this study was conducted in stroke patients, patients deemed appropriate for participating in our study were difficult to recruit from clinical settings. For this reason, we cannot conclude that cervical spine mobilization improved respiratory function and cervical angles until a study using a larger sample size is conducted to confirm our findings. Furthermore, limited evaluation methods were used to accurately examine whether respiratory function and the cervical angles are directly associated with cervical spine mobilization. A phrenic nerve evaluation is additionally needed. The mechanism by which cervical spine mobilization improves respiratory function of stroke patients remains a question that may be answered through further research using a larger number of patients and various evaluation methods.

## 5. Conclusions

The purpose of this study was to investigate the effect of cervical spine mobilization on inspiratory function and cervical angles of stroke patients. Cervical spine mobilization was identified as an intervention technique that can improve inspiratory function of stroke patients. However, cervical spine mobilization did not differ from sham manual treatments except for MIP. This may be due to the NDT program that was applied with it. Therefore, trunk rehabilitation-based NDT program is important for cervical alignment and pulmonary function in stroke patients, and cervical mobilization can reinforce that effect.

## Figures and Tables

**Table 1:** Subject characteristics.

Classification	Experimental Group (*n* = 12)	Control Group (*n* = 12)	*p*-Value ^b^	*p*-Value ^c^
Gender (male/female)	8/4	8/4	0.100	
Paretic side (left/right)	7/5	9/3	0.667	
Pathogenesis (hemorrhages/infarction)	9/3	7/5	0.667	
Disease duration (months) ^a^	13.92 ± 3.32	14.42 ± 3.03		0.662
Age (years) ^a^	66.33 ± 10.58	65.50 ± 9.18		0.795
Weight (kg) ^a^	64.00 ± 8.34	65.30 ± 8.49		0.686
Height (cm) ^a^	165.34 ± 8.56	165.57 ± 8.08		0.729
K-MMSE (point) ^a^	26.25 ± 2.01	26.33 ± 2.06		0.953

^a^ Values are denoted as mean ± SD; ^b^ Chi-square test; ^c^ Mann–Whitney *U* test; K-MMSE: Korean-mini mental state examination; Experimental group: cervical joint mobilization; control group: sham joint mobilization.

**Table 2:** Changes of cervical angle on two intervention groups.

Measure/Group		Baseline Test ^a^	Post Test ^a^	Mean Difference ^a^	Within Group Difference ^b^	Between Group Difference ^c^
CVA (°)	Experimental group	43.27 ± 2.49	47.15 ± 4.08	3.88 ± 3.69	Z = −2.746 *p* = 0.006 *	U = 41.000 *p* = 0.073
	Control group	42.19 ± 2.90	43.88 ± 5.15	1.69 ± 3.11	Z = −1.570 *p* = 0.117	
CRA (°)	Experimental group	147.62 ± 9.47	152.10 ± 5.74	1.41 ± 11.45	Z = −2.197 *p* = 0.028 *	U = 62.000 *p* = 0.564
	Control group	145.65 ± 7.85	148.63 ± 9.70	0.85 ± 8.54	Z = −1.648 *p* = 0.099	

^a^ Values are means ± SD. ^b^ Values of Wilcoxon signed rank test. ^c^ Values of Mann Whitney U test. * Within group difference: significant increase than the baseline test. Experimental group: cervical joint mobilization. Control group: sham joint mobilization. CVA: cranial vertebral angle. CRA: cranial rotation angle.

**Table 3:** Changes of respiratory function on two intervention groups.

Measure/Group		Baseline Test ^a^	Post Test ^a^	Mean Difference ^a^	Within Group Difference ^b^	Between Group Difference ^c^
FVC (*ℓ*)	Experimental group	2.77 ± 0.57	3.13 ± 0.46	0.37 ± 0.29	Z = −2.758 *p* = 0.006 *	U = 44.000 *p* = 0.106
	Control group	2.68 ± 0.53	2.87 ± 0.56	0.19 ± 0.21	Z = −2.601 *p* = 0.009 *	
FEV1 (*ℓ*)	Experimental group	2.34 ± 0.62	2.59 ± 0.54	0.26 ± 0.19	Z = −2.847 *p* = 0.004 *	U = 49.000 *p* = 0.184
	Control group	2.30 ± 0.50	2.46 ± 0.49	0.16 ± 0.15	Z = −2.848 *p* = 0.004 *	
PEF (*ℓ*/min)	Experimental group	260.58 ± 71.24	314.42 ± 61.62	53.83 ± 40.59	Z = −2.847 *p* = 0.004 *	U = 43.000 *p* = 0.094
	Control group	254.08 ± 69.56	283.25 ± 75.52	29.17 ± 28.72	Z = −2758 *p* = 0.006 *	
MIP (mmHg)	Experimental group	39.67 ± 5.91	52.33 ± 3.73	12.67 ± 5.23	Z = −3.065 *p* = 0.002 *	U = 22.000 *p* = 0.003 **
	Control group	39.50 ± 5.28	45.33 ± 3.80	5.83 ± 4.22	Z = −3.070 *p* = 0.002 *	
MEP (mmHg)	Experimental group	53.08 ± 11.08	70.17 ± 11.13	17.08 ± 9.98	Z = −3.061 *p* = 0.002 *	U = 44.000 *p* = 0.106
	Control group	52.50 ± 10.47	63.58 ± 9.63	11.08 ± 5.12	Z = −3.061 *p* = 0.002 *	
Upper chest circumference (cm)	Experimental group	1.61 ± 0.57	2.49 ± 0.84	0.88 ± 0.99	Z = −2.551 *p* = 0.011 *	U = 70.000 *p* = 0.908
	Control group	1.42 ± 0.53	2.19 ± 0.71	0.78 ± 0.87	Z = −2.356 *p* = 0.018 *	
Lower chest circumference (cm)	Experimental group	1.78 ± 0.61	2.76 ± 0.77	0.98 ± 1.06	Z = −2.601 *p* = 0.009 *	U = 67.500 *p* = 0.799
	Control group	1.68 ± 0.44	2.53 ± 0.68	0.85 ± 0.73	Z = −2.551 *p* = 0.011 *	

^a^ Values are means ± SD. ^b^ Values of Wilcoxon signed rank test. ^c^ Values of Mann Whitney *U* test. * Within group difference: significant increase than the baseline test. ** Between group difference: significant increase than the control group. Experimental group: cervical joint mobilization. Control group: sham joint mobilization. FEV1: forced expiratory volume at the 1 s. FVC: forced vital capacity. PEF: peak expiratory flow. MIP: maximal inspiratory pressure. MEP: maximal expiratory pressure.

## Data Availability

Data generated and analyzed during this study are included in this article. Additional data are available from the corresponding author on request.
